# Reduced skin lipid content in obese Japanese women mediated by decreased expression of rate-limiting lipogenic enzymes

**DOI:** 10.1371/journal.pone.0193830

**Published:** 2018-03-08

**Authors:** Yoshiko Horie, Hiroko Makihara, Kazumasa Horikawa, Fumika Takeshige, Ai Ibuki, Toshihiko Satake, Kazunori Yasumura, Jiro Maegawa, Hideaki Mitsui, Kenichi Ohashi, Tomoko Akase

**Affiliations:** 1 Department of Biological Science and Nursing, Yokohama City University Graduate School of Medicine, Yokohama, Kanagawa, Japan; 2 Department of Plastic and Reconstructive Surgery, Yokohama City University Medical Center, Yokohama, Kanagawa, Japan; 3 Plastic and Reconstructive Surgery, Kanagawa Children’s Medical Center, Yokohama, Kanagawa, Japan; 4 Department of Plastic and Reconstructive Surgery, Yokohama City University Graduate School of Medicine, Yokohama, Kanagawa, Japan; 5 Department of Pathology, Yokohama City University Graduate School of Medicine, Yokohama, Kanagawa, Japan; Universite du Quebec a Montreal, CANADA

## Abstract

Skin barrier function is often deficient in obese individuals, but the underlying molecular mechanisms remain unclear. This study investigated how skin structure and lipid metabolism, factors strongly associated with barrier function, differed among 50 Japanese women of greatly varying body mass index (BMI). Subjects receiving breast reconstruction surgery were chosen for analysis to obtain skin samples from the same site. The subjects were classified into two groups, control (BMI < 25 kg/m^2^) and obese (25 kg/m^2^ ≤ BMI < 35 kg/m^2^), according to standards in Japan. Hematoxylin and eosin staining was used to assess skin thickness, Ki-67 immunostaining to examine keratinocyte proliferation, and real-time polymerase chain reaction to measure skin expression levels of genes associated with lipid metabolism. Total lipids, cholesterol, and fatty acids were also measured from these same skin samples. In the obese group, structural changes included epidermal thickening and an increase in the number of Ki-67-positive (proliferating) cells. Both skin cholesterol and fatty acid levels exhibited an “inverted-U” relationship with BMI, suggesting that there is an optimal BMI for peak lipid content and barrier function. Decreased lipid levels at higher BMI were accompanied by downregulated expression of *PPARδ* and other genes related to lipid metabolism, including those encoding acetyl-CoA carboxylase and HMG-CoA reductase, the rate-limiting enzymes for fatty acid and cholesterol synthesis, respectively. Thus, elevated BMI may lead to deficient skin barrier function by suppressing local lipid synthesis.

## Introduction

Obesity is a risk factor for skin disorders[1], including skin diseases characterized by barrier dysfunction such as psoriasis[2] and atopic dermatitis[3]. Transepidermal water loss (TEWL), an indicator of skin barrier function, has been reported to change in obese individuals[4, 5].

Skin structure and lipid content are critical determinants of skin barrier permeability [6]. Lipid levels affect TEWL and the risk of skin barrier disruption[7–10]. In addition, histological changes, including epidermal thickening and keratinocyte proliferation, occur during skin barrier impairment[8, 11, 12]. However, the influence of obesity on these characteristics of barrier dysfunction has not been examined.

Intercellular lipids consist of cholesterol, fatty acids, and ceramide. It is widely known that ceramide contributes to skin barrier function[7]; thus, many previous skin barrier studies focused on ceramide levels[13–16]. For instance, ceramide synthesis decreased in the skin of rats fed a high fat diet[17]. In addition, a cholesterol synthesis inhibitor applied to the skin impaired barrier function[8], while recovery of the barrier was delayed by inhibition of cholesterol synthesis[18]. A decrease in fatty acids has been reported in psoriatic stratum corneum, which is associated with an increase in TEWL[19]. These findings strongly suggest that skin cholesterol and fatty acids are also critical determinants of skin barrier function. Despite this evidence, there are few studies on skin lipid levels or metabolism in obese individuals, a population that generally demonstrates systemic lipid dysregulation as well as skin barrier dysfunction.

The World Health Organization defines obesity as body mass index (BMI) ≥ 30 kg/m^2^ and overweight as BMI ≥ 25 kg/m^2^[20]. However, the associations among BMI, percentage body fat, and body fat distribution differ across ethnic populations, and it was recently suggested that a lower cutoff value may be more appropriate in Asian countries [21, 22]. Accordingly, we recruited Japanese women with a broad range of BMIs and divided them into a control group (BMI < 25 kg/m^2^) and obesity group (25 kg/m^2^ ≤ BMI < 35 kg/m^2^) for comparative analyses of skin structure, lipid contents, and expression of genes involved in lipid metabolism, including genes encoding rate-limiting lipogenic enzymes. Our hypothesis was that skin structure is altered and lipid levels reduced in obese women, thereby accounting for the observed barrier dysfunction in this group.

## Methods

### Subjects

The subjects were Japanese breast cancer patients (20–64 years old) who underwent breast reconstruction surgery via a rectus abdominis flap at a hospital in Yokohama. While chemotherapy can alter skin water content and TEWL of Stage 1–3 breast cancer patients [23], there were no significant differences in chemotherapy regimens between the control and obesity groups. Breast reconstruction was conducted on the premise that disease stage was 1 or 2 and that there was no risk of recurrence or metastasis. Therefore, it was possible to analyze healthy skin at the same site in each individual. Patients with comorbid skin diseases (such as psoriasis and atopic dermatitis), diabetes, or infectious diseases were excluded. Skin samples were collected from the same abdominal region of each subject during surgery. This study was conducted in accordance with the Declaration of Helsinki and with the approval of the Human Genome and Gene Research Ethics Committee of Yokohama City University (A140522018). Written informed consent was obtained from all subjects.

Subjects were divided into two groups, a control group (BMI < 25 kg/m^2^) and obesity group (25 kg/m^2^ ≤ BMI < 35 kg/m^2^) according to the definition of obesity in Japan (BMI ≥ 25 kg/m^2^).

### Hematoxylin and eosin (HE) staining

We evaluated epidermal structure by HE staining (control n = 11, obesity n = 9). Briefly, skin samples were fixed in 10% formalin (Wako Pure Chemical Industries, Ltd., Osaka, Japan) and embedded in paraffin. Then, 5-μm sections were cut, stained with HE (Muto Pure Chemicals Co., Ltd., Tokyo, Japan), and examined under an optical microscope (Olympus Corporation, Tokyo, Japan). Skin thickness of HE-stained histological sections was measured using ImageJ software (https://imagej.nih.gov/ij/). Three sections per individual were used for analysis and epidermis thickness was determined at twenty points per section using the line selection tool.

### Ki-67 staining and calculation of the Mib-1 index

To confirm whether observed changes in epidermal thickness were due to cell proliferation, we performed Ki-67 staining (control n = 11, obesity n = 9). The Ki-67 nuclear antigen is expressed in G1, S, G2, and M phases but is absent in G 0 [24], so Ki-67 immunoreactivity is widely used as a proliferation marker for evaluation of epidermal hypertrophy. For antigen retrieval, the 5-μm paraffin sections were autoclaved (121°C, 20 min) in a solution of 0.1 M citric acid and 0.1 M sodium citrate. Sections were then immersed in methanol containing 0.3% hydrogen peroxide for 15 min to quench endogenous peroxidase activity, washed with phosphate-buffered saline (PBS), and incubated for 60 min at room temperature in 100-fold diluted primary antibody (mouse anti-human Ki-67 antibody; DAKO, Tokyo, Japan). After washing with PBS, the sections were incubated for 30 min at room temperature with a horseradish peroxidase (HRP)-conjugated secondary antibody (goat anti-mouse, anti-rabbit IgG; DAKO). Immunolabeling was visualized with 3,3′-diaminobenzidine (DAB) (DAKO). Sections were then counterstained with hematoxylin and examined under an optical microscope (Olympus Corporation). The total numbers of keratinocytes and Ki-67-positive keratinocytes were counted in five areas of the epidermis per individual. The Mib-1 index was calculated as the percentage of Ki-67-positive cells among total keratinocytes.

### Lipid extraction and quantification

Untreated skin samples were frozen in liquid nitrogen and stored at −80°C until lipid extraction. Adipose tissue was removed from the sections and the remaining skin cut into 0.1-mg pieces. These pieces were homogenized in 50 mM aqueous sodium chloride solution using a Bio Mixer (Nissei Corporation, Tokyo, Japan). Next, a chloroform:methanol (2:1) mixture was added to the lysate in pre-weighed centrifuge tubes, and the suspension centrifuged (2,000 ×*g*, 4°C, 10 min). The bottom organic layer was removed and allowed to dry overnight. The total lipid weight was calculated by weighing the tubes and subtracting the empty tube weight. Total lipid extracts were dissolved in octylphenol ethoxylate (Wako Pure Chemical Industries, Ltd.) adjusted to 20% (v/v) with 2-propanol (Wako Pure Chemical Industries, Ltd.), and the concentrations of cutaneous cholesterol and fatty acids determined using commercial kits (Cholesterol E-Test, Wako Pure Chemical Industries, Ltd. And NEFA C-Test, Wako Pure Chemical Industries, Ltd., respectively).

### Real-time polymerase chain reaction (RT-PCR)

Skin samples were frozen in liquid nitrogen and stored at −80°C until RNA extraction. Samples were placed in 1 ml of total RNA extraction solution (RNAiso, Takara Bio Inc., Shiga, Japan) and homogenized using a Bio Mixer (Nissei Corporation). Next, chloroform was added (200 μl) and the homogenate/chloroform mixture centrifuged (10,000 ×*g*, 4°C, 15 min). The clear supernatant was collected, mixed with 2-propanol (500 μl), and allowed to stand at room temperature for 10 min. This mixture was then centrifuged (10,000 ×*g*, 4°C, 15 min), the aqueous layer discarded, and 70% ethanol (1 ml) added to the pellet. The new suspension was centrifuged (4,000 ×*g*, 4°C, 5 min), and the aqueous layer again discarded. The pellet (total RNA sample) was air dried and dissolved in ribonuclease (RNase)-free water (QIAGEN GmbH, Hilden, Germany). cDNA was synthesized from total RNA using the Primescript RT reagent kit with genomic deoxyribonucleic acid (gDNA) Eraser (Takara Bio Inc.). Gene expression levels were estimated by RT-PCR using SYBR Premix Ex Taq II (Takara Bio Inc.) and the CFX96 real-time analysis system (Bio-Rad Laboratories, Inc., Hercules, CA, USA). For quantitative analysis, samples were heat denatured at 95°C for 10 s, and target gene fragments were amplified by 45 cycles of 95°C (5 s), 57°C (10 s), and 72°C (10 s). The β-actin gene (*ACTB*) was used as the internal control, and the calibration curve method was used to measure the expression levels of genes related to cholesterol metabolism [3-hydroxy-3-methylglutaryl-CoA reductase (*HMGCR*), sterol regulatory element-binding protein-2 (*SREBP-2*), low-density lipoprotein receptor (*LDLR*), and liver X receptor (*LXRα*)], fatty acids metabolism [fatty acid synthase (*FAS*), acetyl-CoA carboxylase-1 (*ACC-1*), stearoyl-CoA desaturase-1 (*SCD-1*), sterol regulatory element-binding protein-1c (*SREBP-1c*), and carnitine palmitoyltransferase-1 alpha (*CPT-1α*)], and lipid metabolism [peroxisome proliferator-activated receptor alpha (*PPARα*) and peroxisome proliferator-activated receptor delta (*PPARδ*)]. Primer sequences are shown in S1 Table.

### Statistical analysis

All data are expressed as mean ± SD. Continuous data were compared between groups by unpaired Student’s *t*-tests and categorical data by Pearson’s χ^2^ test or Fisher’s exact test. Spearman’s rank correlation was used to examine associations of BMI with skin lipid concentrations and expression levels of genes involved in lipid metabolism. SPSS software version 22 (IBM Corporation, Armonk, NY, USA) was used for all statistical analyses. A *p*-value < 0.05 (two-tailed) was considered statistically significant.

## Results

### Demographic and clinical characteristics of subjects

The study included 50 subjects, of which 39 were assigned to the control group and 11 to the obesity group based on a BMI cut-off of 25 kg/m^2^. Mean BMI differed significantly between the control and obesity groups (22.3 ± 1.7 vs. 27.2 ± 2.2) but there were no other significant differences in clinical or demographic characteristics between groups (Table 1).

**Table 1:** Patient characteristics.

	Control (n = 39)	Obesity (n = 11)	*p*
BMI (kg/m^2^)^d^	22.3±1.7	27.2±2.2	<0.01^a^
Age (year)^d^	50.4±7.3	51.9±6.3	0.54^a^
**Clinical history**			
Hypertension^e^	8 (20.5)	0 (0.0)	0.12^c^
Dyslipidemia^e^	2 (5.1)	0 (0.0)	0.61^c^
Diabetes mellitus^e^	0 (0.0)	0 (0.0)	-
Dermatosis^e^	0 (0.0)	0 (0.0)	-
Smoking history^e^	10 (25.6)	3 (27.3)	0.60^c^
Chemotherapy^e^	16 (41.0)	5 (45.5)	0.53^c^
Radiation therapy^e^	8 (20.5)	1 (9.1)	0.36^c^

N = 50,

^a^ Student’s t test,

^b^ χ2 test,

^c^ Fisher’s exact test,

^d^ Average±SD, en (%)

Demographic and clinical characteristics of the control group (BMI < 25 kg/m^2^, n = 39) and the obesity group (25 kg/m^2^ ≤ BMI < 35 kg/m^2^, n = 11).

### Structural changes in the epidermis associated with obesity

**Fig 1:**
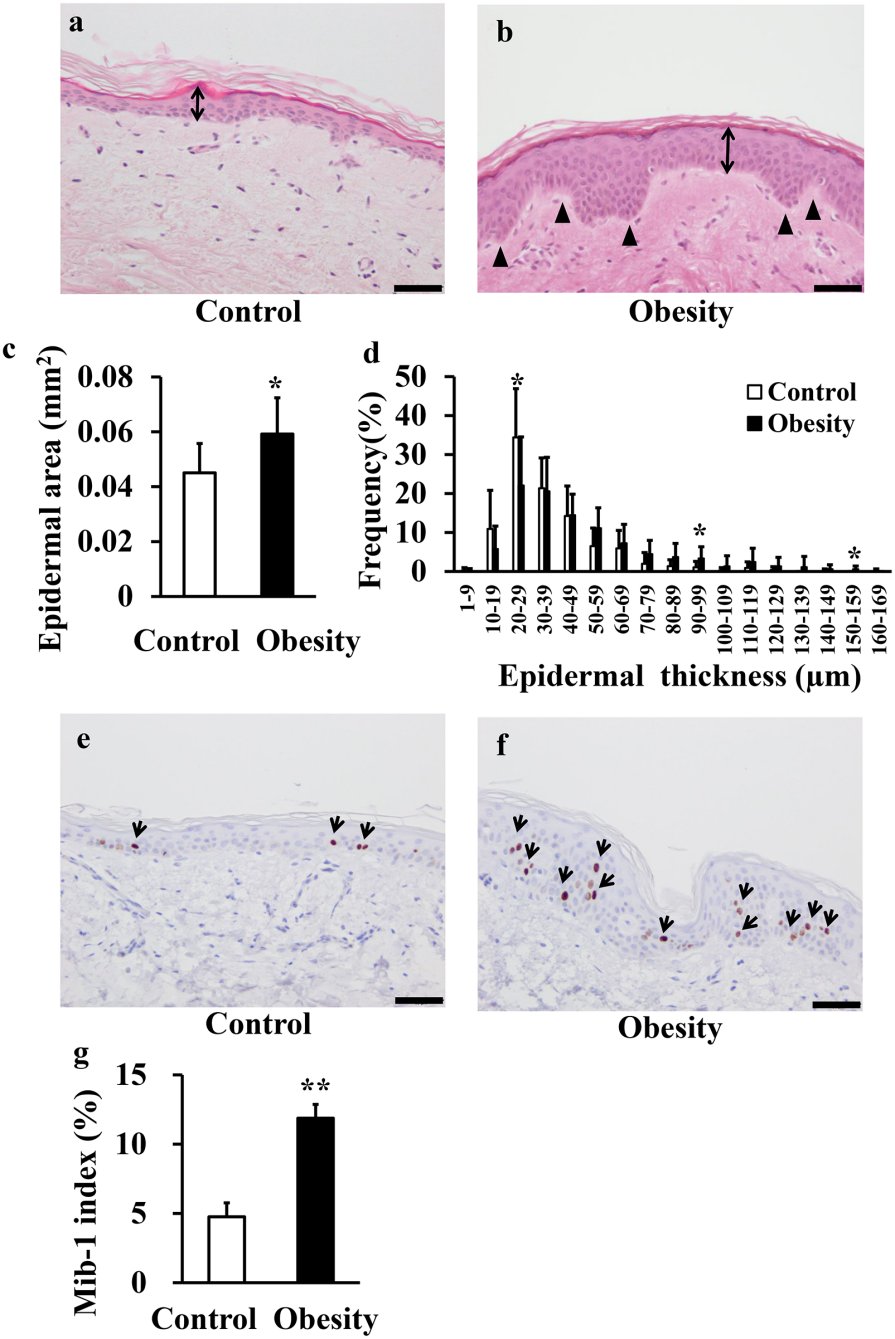
Skin histology in control and obesity groups. (a, b) HE staining of skin samples from the control group (a. BMI < 25 kg/m^2^) and the obesity group (b. 25 ≤ BMI < 35 kg/m^2^) (bar = 50 μm). The arrows (↕) indicate epidermal thickness and the arrowheads (▲) show the unevenness of epidermal thickness. (c) Epidermal area (mm^2^) in the control group and the obesity group. (d) Frequency histogram of epidermal thickness in the control group and the obesity group. (e, f) Ki-67 staining in the control group (e) and obesity group (f) (bar = 50 μm). Reddish brown cells are Ki-67-positive, suggesting proliferation (↑: arrows). (g) Percentages of Ki-67-positive cells (Mib-1 index) in the control group and the obesity group. Data are presented as mean ± SD. Statistical comparisons between the groups are performed using unpaired Student’s *t*-test (*p<0.05, **p<0.01).

Examples of HE-stained skin samples from control (BMI < 25 kg/m^2^) and obesity (25 ≤ BMI < 35 kg/m^2^) groups are shown in Fig 1a and 1b, respectively. The epidermis was thickener and more uneven in the obesity group compared to the control group. Additionally, epidermal area was significantly greater in the obesity group than the control group (Fig 1c). The distribution of epidermal thickness was shifted to higher values in the obesity group (Fig 1d). For instance, the proportion 20–29 μm thick was significantly reduced and the proportions 90–99 μm and 150–159 μm thick were significantly greater in the obesity group compared to the control group.

### Evaluation of cell proliferation

In the control group, Ki-67-positive cells were observed only in the stratum basal and suprabasal layers of the epidermis (Fig 1e). In the obesity group, however, Ki-67-positive cells were also observed in the middle layer of the epidermis, and total numbers were greater than in the control group (Fig 1f). Moreover, the proportion of Ki-67-positive keratinocytes (Mib-1index) was significantly higher in the obesity group than the control group (Fig 1g).

### Quantification of cholesterol and fatty acids

**Fig 2:**
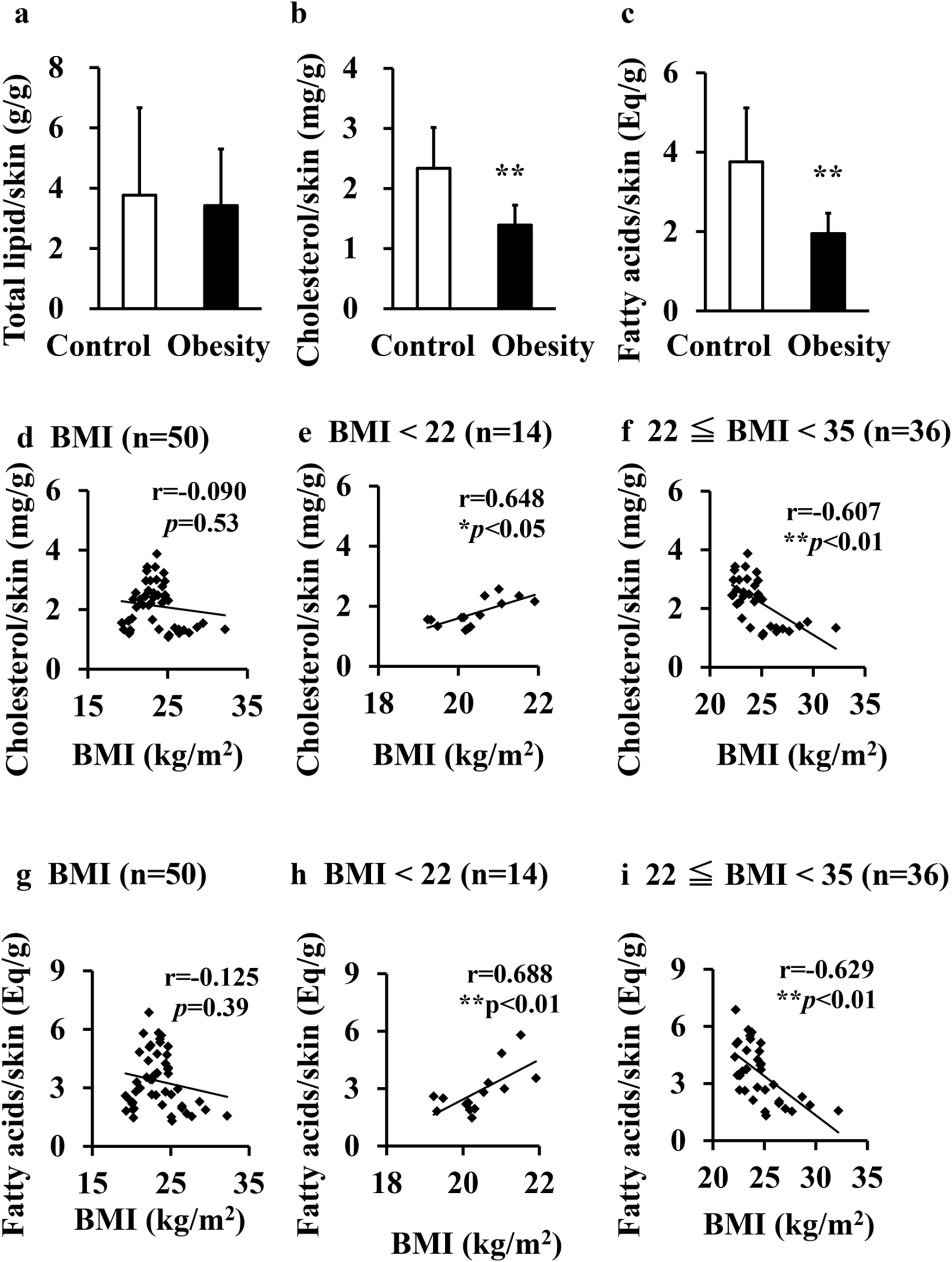
Levels of each lipid in the skin. Total lipids (a), cholesterol (b), and fatty acids (c) in the skin are compared between the control group (BMI < 25 kg/m^2^, n = 39) and obesity group (25 kg/m^2^ ≤ BMI < 35 kg/m^2^, n = 11). Data are presented as mean ± SD. Statistical comparisons between the groups are performed using the unpaired Student’s *t*-test (**p < 0.01). (d-i) Spearman’s rank correlation coefficients (r) between BMI and each lipid for all subjects (d, g), subjects with BMI < 22 kg/m^2^ (e, h), and subjects with BMI from 22–35 kg/m^2^ (f, i) (**p < 0.01).

Total skin lipid concentration did not differ between groups (Fig 2a). However, cholesterol (Fig 2b) and fatty acid (Fig 2c) levels were significantly lower in skin samples of the obesity group compared to the control group.

Both cholesterol and fatty acid levels (Fig 2d and 2g) exhibited an “inverted-U" shape relationship with BMI. Based on epidemiological data, disease prevalence and mortality rate are lowest in Japanese women with BMI = 22 kg/m^2^ [25, 26], so BMI = 22 kg/m^2^ was used as the cut-off to examine the relationships between skin lipids and both low and high BMI. Cholesterol and fatty acid levels were negatively correlated with BMI in subjects with high BMI (≥22 kg/m^2^ but < 35 kg/m^2^) (Fig 2f and 2i) but positively correlated with BMI in subjects with low BMI (< 22 kg/m^2^) (Fig 2e and 2h). In contrast, there were no significant correlations between lipid levels and age (Total lipid: r = 0.173 *p* = 0.23, Cholesterol: r = −0.136 *p* = 0.25, Fatty acids: r = −0.087 *p* = 0.57).

### Expression of genes related to lipid metabolism

To investigate the underlying mechanisms contributing to these BMI-dependent changes in skin lipid concentrations, we evaluated the expression levels of genes related to lipid metabolism (Table 2). The expression levels of *PPARα*, *PPARδ*, *HMGCR* (encoding the rate-limiting enzyme for cholesterol synthesis), *SREBP-2* (encoding a transcription factor that regulates cholesterol metabolism), *FAS* (encoding a rate-limiting enzyme in fatty acid synthesis), and *ACC-1* (encoding another rate-limiting enzyme in fatty acid synthesis) were significantly lower in the obesity group than the control group. Next, the correlation between BMI and each gene expression level was examined (Table 3). No gene was significantly negatively correlated with BMI in subject with high BMI (≥ 22 kg/m^2^ but < 35 kg/m^2^) but positively correlated with BMI in subject with low BMI (< 22 kg/m^2^), like lipid levels. However, expression levels of *PPARδ*, *HMGCR*, and *ACC-1* were negatively correlated with BMI in the BMI range ≥ 22 kg/m^2^ to < 35 kg/m^2^. Expression of *LDLR*, which functions in cellular cholesterol uptake and transport, did not correlate with BMI, while the expression of *LXRα*, which causes cholesterol efflux, was significantly and negatively correlated with BMI when < 22 kg/m^2^.

**Table 2:** Expression levels of lipid metabolism gene in the control and the obesity group.

Gene	Control (n = 39)	Obesity (n = 11)	*p*
**Lipids metabolism**			
*PPARα*	1.0±0.4	0.7±0.3	<0.05*
*PPARδ*	1.0±0.5	0.6±0.3	<0.01**
**Cholesterol metabolism**			
*HMGCR*	1.0±0.5	0.4±0.2	<0.01**
*SREBP-2*	1.0±0.4	0.6±0.3	<0.01**
*LDLR*	1.0±0.4	0.8±0.4	0.07
*LXRα*	1.0±0.3	1.0±0.3	0.83
**Fatty acids metabolism**			
*SREBP-1c*	1.0±0.4	0.8±0.4	0.12
*FAS*	1.0±0.6	0.6±0.3	<0.05*
*ACC-1*	1.0±0.5	0.5±0.2	<0.01**
*SCD-1*	1.0±0.7	0.9±0.5	0.54
*CPT-1α*	1.0±0.4	0.8±0.4	0.17

Expression levels of lipid metabolism-related genes in skin samples of the control group and the obesity group. Data are presented as mean ± SD. Statistical comparisons between the groups are performed by unpaired Student’s *t*-test (*p<0.05, **p<0.01).

**Table 3:** Correlation between gene expression and BMI.

	all subjects		BMI <22		22 ≦ BMI < 35	
Gene	r	*p*	r	*p*	r	*p*
**Lipids metabolism**						
*PPARα*	0.003	0.98	-0.363	0.20	-0.154	0.37
*PPARδ*	-0.276	0.06	0.332	0.25	-0.426	<0.05*
Cholesterol metabolism						
*HMGCR*	-0.183	0.20	0.305	0.29	-0.602	<0.01**
*SREBP-2*	-0.230	0.11	-0.196	0.50	-0.310	0.07
*LDLR*	-0.160	0.27	-0.209	0.49	-0.264	0.12
*LXRα*	0.039	0.79	-0.705	<0.01**	0.010	0.95
Fatty acids metabolism						
*SREBP-1c*	-0.017	0.91	-0.068	0.82	-0.065	0.71
*FAS*	-0.097	0.52	0.020	0.95	-0.204	0.26
*ACC-1*	-0.045	0.75	0.336	0.24	-0.559	<0.01**
*SCD-1*	0.294	<0.05*	0.200	0.49	-0.027	0.88
*CPT-1α*	-0.180	0.22	-0.349	0.22	-0.148	0.40

Spearman’s rank correlation coefficients (r) of BMI with the expression levels of lipid metabolism-related genes (*p<0.05, **p < 0.01).

### Correlations between lipid-related gene expression and skin lipids contents

To confirm that these alterations in skin expression levels of lipid metabolism-related genes contribute to group differences in skin lipid levels, we evaluated the correlations between each skin lipid concentration and gene expression level among all subjects. The expression levels of *PPARδ* and *HMGCR* were positively and significantly correlated with skin cholesterol level, and the expression levels of *PPARδ* and *ACC-1* were positively and significantly correlated with skin fatty acid level (Table 4).

**Table 4:** Correlation between gene expression and the amounts of each lipid in the skin.

Cholesterol			Fatty acids		
Gene	r	*p*	Gene	r	*p*
*PPARα*	0.182	0.21	*PPARα*	0.110	0.45
*PPARδ*	0.323	<0.05*	*PPARδ*	0.434	<0.01**
*HMGCR*	0.476	<0.01**	*SREBP-1c*	-0.053	0.72
*SREBP-2*	0.150	0.30	*FAS*	0.052	0.73
*LDLR*	0.045	0.76	*ACC-1*	0.494	<0.01**
*LXRα*	-0.036	0.81	*SCD-1*	0.053	0.72
			*CPT-1α*	-0.105	0.47

Spearman’s rank correlation coefficients (r) for lipid metabolism-related gene expression levels versus lipid levels in skin samples from all subjects (*p<0.05, **p < 0.01).

## Discussion

This is the first study to demonstrate changes in epidermal structure and decreased cholesterol and fatty acid levels in the skin of obese adults. Our study suggests that these skin lipid reductions are mediated by decreased expression levels of multiple genes related to lipid metabolism, including the rate-limiting enzymes for fatty acid and cholesterol synthesis. The relationship between BMI and skin lipid levels exhibited an inverted-U shape, indicating that peak lipid content and skin barrier function depend on an optimal BMI range (around 22 kg/m^2^). Thus, adults with aberrantly low BMI (much less than 22 kg/m^2^) or high BMI (≥25 kg/m^2)^ appear to be at greater risk of skin disorders associated with reduced barrier function due to dysregulation of skin lipid metabolism.

The skin of obese Japanese women also exhibited uneven epidermal thickening, with ectopic proliferation of keratinocytes and greater overall keratinocyte numbers as revealed by immunohistological analysis. These histological findings are consistent with the skin barrier disruption model[11, 12, 27]. In obese people, epidermal thickening usually occurs on the soles of the feet and at other sites on the skin where pressure and irritation cause inflammation[28]. Increased epidermal turnover is also induced by external stimuli, including ultraviolet rays[29]. In this study, however, epidermal thickening also developed in an area not regularly subjected to pressure or external irritation (abdominal region). In addition, the expression levels of the inflammation markers *TNF-α* and *IL-6* in skin were not correlated with BMI (S2 Table). Thus, the structural and molecular changes observed in the skin of obese women appear to be independent of inflammation.

Skin barrier disruption due to increased TEWL has also been reported with decreased levels of individual lipids (i.e., cholesterol and fatty acids)[8, 30]. Our results indicated that obesity decreases skin cholesterol and fatty acid levels, suggesting that low levels of these lipids contribute to skin barrier disruption observed in obesity[4, 5]. Cholesterol and fatty acid levels were negatively correlated with BMI in the range above the optimal value (i.e., > 22 kg/m^2^ to 35 kg/m^2^) and positively correlated with BMI below this cut-off (< 22 kg/m^2^). Further, skin cholesterol and fatty acid levels exhibited as inverted-U shape, like disease prevalence [25, 26].

Thus, skin lipid levels appear to rise with BMI in low-weight women and decrease with BMI in overweight/obese women.

In this study, skin cholesterol was measured as total cholesterol, which may include 7-dehydrocholesterol, a vitamin D precursor[31, 32]. It was reported that obese individuals have a reduced capacity to synthesize vitamin D after exposure to ultraviolet radiation, and so are at increased risk of vitamin D deficiency. Therefore, reduced skin cholesterol in these obese subjects may be associated with vitamin D deficiency and related diseases.

Skin cholesterol and fatty acid levels were positively and significantly correlated with the expression levels of *HMGCR* and *ACC-1*, respectively, in the BMI range ≥ 22 kg/m^2^ to < 35 kg/m^2^. Yamane et al. reported that a high fat diet decreased the ceramide content in the skin of rats[17]. Transcription of *HMGCR* is promoted by binding of SREBP-2 protein to a specific region of the *HMGCR* promoter. In our study, *SREBP-2* expression in the skin tended to be negatively correlated with BMI above the cut-off (>22 kg/m^2^) and so was significantly lower in the obesity group (BMI ≥ 25 kg/m^2^) than the control group (BMI < 25 kg/m^2^). This result suggests that skin *HMGCR* transcription decreases in high-BMI Japanese women as a result of decreased skin *SREBP-2* expression, which in turn reduces cholesterol synthesis. In contrast, skin cholesterol content was positively correlated with BMI below the cut-off (< 22 kg/m^2^) while *LXRα* expression was negatively correlated with BMI below 22 kg/m^2^; however, no relationships were observed between BMI and expression levels of *HMGCR* and *SREBP-2* in these low-weight women. These results suggest that skin cholesterol is regulated by different sets of genes below and above BMI = 22 kg/m^2^ (either for synthesis or for excretion). Expression of lipid metabolism-related genes is regulated by nuclear receptor *PPARs*. In the skin of obese women, the amounts of cholesterol and free fatty acids were positively correlated with *PPARδ* expression, indicating that *PPARδ* downregulation at BMI > 22 kg/m^2^ reduces the amounts of both skin lipid types. Although additional research on the underlying molecular mechanisms is necessary, we speculate that, unlike lipid regulation in liver and adipose tissue, lipid syntheses in skin is suppressed by obesity due to downregulation of lipogenic genes. However, skin barrier function depends on multiple factors in addition to lipid content. Future research is needed to assess how obesity alters epithelial integrity and other components of the skin barrier.

## Conclusion

In conclusion, obesity in Japanese women is associated with epidermal thickening and reduced skin cholesterol and fatty acid levels, suggesting impaired skin barrier function. Further, lipid levels exhibited an inverted-U relationship with BMI, suggesting that there is an optimal BMI around 22 kg/m^2^ for peak lipid content and barrier function. Skin lipid reduction in obese individuals was associated with downregulation of *PPARδ* expression and concomitant downregulation of *HMGCR* and *ACC-1*, genes encoding rate-limiting enzymes for cholesterol and fatty acid synthesis, respectively. These results suggest the that suboptimal BMI is associated with impaired skin barrier function due to low skin cholesterol and fatty acid levels. Furthermore, our results may have implications for treatment of skin disease associated with barrier dysfunction in obese patients.

## Supporting information

S1 Table Primer sequences outlined in the 5'-3' direction. (DOCX)

S2 Table Correlations between pro-inflammatory genes and BMI. (DOCX)
