# Supplementary material for: Reduced skin lipid content in obese Japanese women mediated by decreased expression of rate-limiting lipogenic enzymes
Source: PLoS One. 2018 Mar 8;13(3):e0193830. doi: 10.1371/journal.pone.0193830 (PMC5843255; doi:10.1371/journal.pone.0193830)
Supplement: S1 Table — (DOCX) [file pone.0193830.s001.docx]

**Supplemental Table S1. Primer sequences outlined in the 5'-3' direction**

| Gene | Sequences | Accession number |
| --- | --- | --- |
| *HMGCR* | Forward: 5’-TGATTGACCTTTCCAGAGCAAG-3’ | NM_001130996.1 |
|  | Reverse: 5’-CTAAAATTGCCATTCCACGAGC-3’ | NM_000859.2 |
| [*SREBP-1c*](http://www.kyoto-u.ac.jp/static/ja/news_data/h/h1/news6/2013_1/131203_2.htm#b) | Forward: 5’-TCAGCGAGGCGGCTTTGGAGCAG-3’ | NM_004176.4 |
|  | Reverse: 5’-CATGTCTTCGATGTCGGTCAG-3’ | NM_001005291.2 |
| [*SREBP-2*](http://www.kyoto-u.ac.jp/static/ja/news_data/h/h1/news6/2013_1/131203_2.htm#b) | Forward: 5’-TGGCTTCTCTCCCTACTCCA-3’ | NM_004599.3 |
|  | Reverse: 5’-GAGAGGCACAGGAAGGTGAG-3’ | NR_103834.1 |
| *PPARα* | Forward: 5’-CAGAACAAGGAGGCGGAGGTC-3’ | NM_001001928.2 |
|  | Reverse: 5’-TTCAGGTCCAAGTTTGCGAAGC-3’ | NM_005036.4 |
| *PPARδ* | Forward: 5’-CTCTATCGTCAACAAGGACG-3’ | NM_001171820.1 |
|  | Reverse: 5’-GTCTTCTTGATCCGCTGCAT-3’ | NM_001171819.1 |
|  |  | NM_001171818.1 |
|  |  | NM_006238.4 |
|  |  | NM_177435.2 |
| *LDLR* | Forward: 5’-GCTTGTCTGTCACCTGCAAA-3’ | NM_001195803.1 |
|  | Reverse: 5’-AACTGCCGAGAGATGCACTT-3’ | NM_001195800.1 |
|  |  | NM_001195799.1 |
|  |  | NM_001195798.1 |
|  |  | NM_000527.4 |
| *LXRα* | Forward: 5’-GGAGGTACAACCCTGGGAGT-3’ | NM_001251935.1 |
|  | Reverse: 5’-AGCAATGAGCAAGGCAAACT-3’ | NM_001251934.1 |
|  |  | NM_001130102.2 |
|  |  | NM_001130101.2 |
|  |  | NM_005693.3 |
| *CPT-1α* | Forward: 5’-CGCTACTCCCTGAAAGTG-3’ | NM_001031847.2 |
|  | Reverse: 5’-CTTGACCATACCCATCCAG-3’ | NM_001876.3 |
| *FAS* | Forward: 5’-AACTCCAAGGACACAGTCACCAT-3’ | NM_004104.4 |
|  | Reverse: 5’-CAGCTGCTCCACGAACTCAA-3’ |  |
| *ACC-1* | Forward: 5’-TTAACAGCTGTGGAGTCTGGCTGT-3’ | NM_198836.2 |
|  | Reverse: 5’-AACACTCGATGGAGTTTCTCGCCT-3’ | NM_198834.2 |
|  |  | NM_198839.2 |
|  |  | NM_198838.1 |
|  |  | NM_198837.1 |
| *SCD-1* | Forward: 5’-CTCCACTGCTGGACATGAGA-3’ | NM_005063.4 |
|  | Reverse: 5’-AATGAGTGAAGGGGCACAAC-3’ |  |
| *SPT-1* | Forward: 5’-GCGCGCTACTTGGAGAAAGA-3’ | NM_001281303.1 |
|  | Reverse: 5’-TGTTCCACCGTGACCACAAC-3’ | NM_006415.3 |
| *TNF-α* | Forward: 5’-TTGAGGGTTTGCTACAACATGGG-3’ | NM_000594.3 |
|  | Reverse: 5’-GCTGCACTTTGGAGTGATCG-3’ |  |
| *IL-6* | Forward: 5’-TTTTGTACTCATCTGCACAGC-3’ | NM_001318095.1 |
|  | Reverse: 5’-GGATTCAATGAGGAGACTTGC-3’ | NM_000600.4 |
| *ACTB* | Forward: 5’-AGAGCTACGAGCTGCCTGAC-3’ | NM_001101.3 |
|  | Reverse: 5’-AGCACTGTGTTGGCGTACAG-3’ |  |
