# Supplementary material for: Reduced skin lipid content in obese Japanese women mediated by decreased expression of rate-limiting lipogenic enzymes
Source: PLoS One. 2018 Mar 8;13(3):e0193830. doi: 10.1371/journal.pone.0193830 (PMC5843255; doi:10.1371/journal.pone.0193830)
Supplement: S2 Table — (DOCX) [file pone.0193830.s002.docx]

**Supplemental Table S2. Correlation between gene expression related to inflammation and BMI.**

|  | all subjects | | BMI <22 | | 22 ≦ BMI < 35 | |
| --- | --- | --- | --- | --- | --- | --- |
| Gene | r | *p* | r | *p* | r | *p* |
| *TNF-α* | 0.013 | 0.93 | 0.511 | 0.05 | -0.230 | 0.18 |
| *IL-6* | -0.130 | 0.42 | -0.327 | 0.33 | -0.018 | 0.92 |

r: correlation coefficient. *p<0.05, **p<0.01
